# Comparison of the Administration Route of Stem Cell Therapy for Ischemic Stroke: A Systematic Review and Meta-Analysis of the Clinical Outcomes and Safety

**DOI:** 10.3390/jcm12072735

**Published:** 2023-04-06

**Authors:** Asra Al Fauzi, Ahmad Muslim Hidayat Thamrin, Andhika Tomy Permana, I. G. M. Aswin R. Ranuh, Hanik Badriyah Hidayati, Muhammad Hamdan, Joni Wahyuhadi, Nur Setiawan Suroto, Pudji Lestari, Poodipedi Sarat Chandra

**Affiliations:** 1 Department of Neurosurgery, Faculty of Medicine, Universitas Airlangga, Dr. Soetomo General Academic Hospital, Surabaya 60286, Indonesia; 2 Department of Neurology, Faculty of Medicine, Universitas Airlangga, Dr. Soetomo General Academic Hospital, Surabaya 60286, Indonesia; 3 Department of Public Health and Preventive Medicine, Faculty of Medicine, Universitas Airlangga, Dr. Soetomo General Academic Hospital, Surabaya 60286, Indonesia; 4 Department of Neurosurgery, All India Institute of Medical Sciences, New Delhi 110608, India

**Keywords:** stem cell therapy, ischemic stroke, administration route, outcome, adverse events

## Abstract

Stem cell treatment is emerging as an appealing alternative for stroke patients, but there still needs to be an agreement on the protocols in place, including the route of administration. This systematic review aimed to assess the efficacy and safety of the administration routes of stem cell treatment for ischemic stroke. A systematic review was performed according to the Preferred Reporting Items for Systematic Reviews and Meta-Analysis (PRISMA) guidelines. A comprehensive literature search was undertaken using the PubMed, Scopus, and Cochrane databases. A total of 21 publications on stem cell therapy for ischemic stroke were included. Efficacy outcomes were measured using the National Institutes of Health Stroke Scale (NIHSS), the modified Rankin Scale (mRS), and the Barthel index (BI). Intracerebral administration showed a better outcome than other routes, but a greater number of adverse events followed due to its invasiveness. Adverse events were shown to be related to the natural history of stroke not to the treatment. However, further investigation is required, since studies have yet to compare the different administration methods directly.

## 1. Introduction

Stroke, one of the top causes of death worldwide, continues to be a significant costly drain on global health resources [1]. Stroke is estimated to be responsible for around 140,000 fatalities yearly in the United States [2]. Most strokes are ischemic in nature, representing about 87% of all instances in the US, making it the main focus of stroke research [2]. Even though 80% of stroke patients survive for one year after an incident, more than 10% of patients have long-term disabilities [3].

The increase in survival and reduction in sequelae following ischemic stroke may be partially explained by the acute-phase delivery of thrombolytic therapies [4]. The only approved therapy for acute stroke is intravenous recombinant tissue Plasminogen Activator (tPA), which has a limited time window of only 4.5 h. Additionally, there is a strict patient criteria for receiving urgent endovascular therapy and the benefits are uncertain [5]. Due to this failure to achieve the anticipated outcomes, it is only natural that new therapeutic tactics with a longer time frame and less invasive approaches are urgently needed.

Numerous studies have demonstrated the efficacy of stem cell treatment in restoring functional ability in stroke patients [6]. Aside from its potential to stimulate endogenous reparative mechanisms without replacing the injured cerebral tissue [7], it has also been shown to promote immunomodulation and neuronal, vascular, and glial remodeling. Intravenous injection is one option for the administration of neurotrophic substances, including vascular endothelial growth factor (VEGF), insulin-like growth factor (IGF), and brain-derived neurotrophic factor [8]. The type and source of stem cell to be administered (e.g., mesenchymal stem cell, bone marrow mononuclear cell, or neural stem cell), the route of administration (e.g., intravenous, intra-arterial, or intracerebral), and the time interval between stroke onset and administration (days to months), are all said to explain the varying results [9]. Clinical trials of stem cell therapy in stroke patients have demonstrated that the therapy is feasible, safe, and promotes recovery in ischemic strokes [10]. Since only a few studies have examined this treatment’s clinical outcome, effectiveness, and safety, as related to its route of administration, this review is mainly to determine the treatment’s clinical benefits and adverse events according to the treatment’s safety [11,12,13,14].

## 2. Materials and Methods

### 2.1. Eligibility Criteria

This review includes full-text cohort studies and clinical trials on adult ischemic stroke patients (acute, subacute, or chronic) who received stem cell therapy via intracerebral, intraventricular, subarachnoid, intra-arterial, intravenous, intraperitoneal, or intranasal administration. Reviews, unpublished articles, letters to the editor, abstracts, and studies not written in English were excluded.

### 2.2. Type of Outcome Measurements

Clinical outcomes were measured using the modified Rankin scale (mRS), the National Institute of Health Stroke Scale (NIHSS), and the Barthel index (BI). The adverse events (AE) and severe adverse events (SAE) of stem cell administration routes were analyzed to identify the safety of each treatment.

### 2.3. Search Methods and Identification of Studies

#### 2.3.1. Information Sources

**Figure 1:**
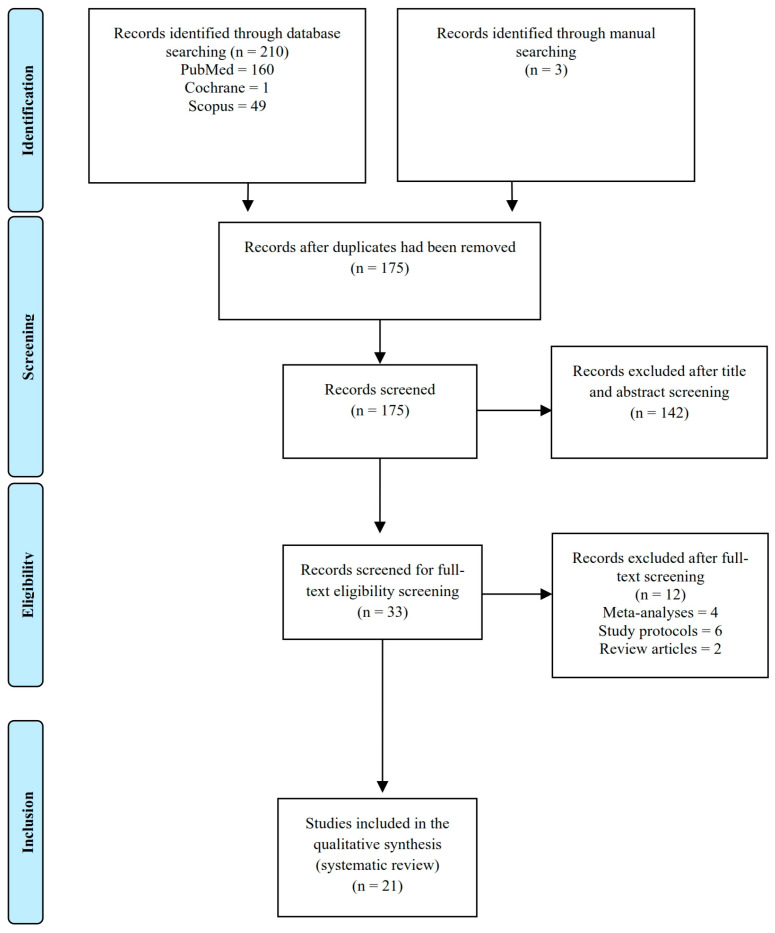
Preferred Reporting Items for Systematic Reviews and Meta-Analysis (PRISMA) guidelines flowchart.

This systematic review was conducted based on Preferred Reporting Items for Systematic Reviews and Meta-Analysis (PRISMA) guidelines [15]. The articles, which were published between 2010 and 2022, were acquired by searching the PubMed, Scopus, Cochrane, and other electronic database sources (Google Scholar) on 31 March 2022. We applied language restrictions to our search so that only articles written in English were selected (Figure 1).

#### 2.3.2. Search Protocol

The study questions were formed using the patient/population, intervention, comparison, outcomes, and study design (PICOS) model (Table 1). The following search string was used to search all trial registers and databases: stem cell therapy AND (ischemic stroke OR ischemic brain) AND (intracerebral OR intraventricular OR subarachnoid OR intra-arterial OR intravenous OR intraperitoneal OR intranasal) AND (functional outcome OR mRS OR NIHSS OR BI).

### 2.4. Data Collection and Analysis

The included studies were screened based on their titles and abstracts. Full-text articles, including RCTs and cohort studies, fulfilling the eligibility criteria were then assessed by each author. The details regarding the causes of exclusion were noted and reported. The included studies are summarized in Table 2.

### 2.5. Data Extraction and Management

Four authors independently extracted data, including each article’s patient characteristics, treatments, research quality, and therapeutic results. Details regarding the author, year of publication, study design, treatment details, and functional outcome based on the predetermined parameters were summarized for the qualitative analysis. We extracted the mean difference of outcome in each arm for continuous outcomes (mean difference of NIHSS, mRS, and BI after 6, 12, and 24 months). The four review authors entered all data into Review Manager (RevMan) software, version 5.4 [15]. Unfortunately, the literature search was carried out before we were registered to any of the systematic review registries.

### 2.6. Risk of Bias Analysis

Each author independently assessed the risk of bias in each study using Risk of Bias in Nonrandomized Intervention Studies (ROBINS-I) for nonrandomized studies and Risk of Bias 2 (RoB 2) for randomized studies [16]. The results of each interpreter’s assessment were then discussed by all of the authors. A risk-of-bias table and a summary of the bias were used. These showed normal distribution results with some acceptable deviations, and thus the eligibility of the literature was high [17,18].

**Table 2:** Summary of the Studies Included in the Systematic Review.

Author(s)	Study Design	Age (years)	Stroke Territory	Sample Size	Type of Graft	Number of Transplanted Cells	Route of Administration	Functional Outcome												Notes
								Baseline			6 Months			12 Months			24 Months			
								NIHSS	mRS	BI	NIHSS	mRS	BI	NIHSS	mRS	BI	NIHSS	mRS	BI	
Lee et al. (2010) [19]	RCT	64.6 ± 13.6	52 MCA	36 control	Autologous bone marrow MSC	50 × 10^6^	Intravenous	10.63 ± 3.00	4.80 ± 0.50	n/a	n/a	n/a	n/a	n/a	n/a	n/a	n/a	n/a	n/a	Improvement in the early post-treatment phase, no improvement later on
				16 treatment				10.17 ± 3.60	4.40 ± 0.90	n/a	n/a	n/a	n/a	n/a	n/a	n/a	n/a	n/a	n/a	
Honmou et al. (2011) [20]	Non-RCT	60.5 (41–73)	4 MCA 6 ICA 2 Lacunar	12 treatment	Autologous bone marrow-derived MSC	120 (60–160) × 10^6^	Intravenous	8.25 ± 5.55	n/a	n/a	n/a	n/a	n/a	1.58 ± 2.02	n/a	n/a	n/a	n/a	n/a	Significant improvement
Bhasin et al. (2012) [21]	Non-RCT	46.58 ± 10.99	24 MCA	12 control	Autologous bone marrow–derived MSC	55 (50–58) × 10^6^	Intravenous	n/a	n/a	49.92 ± 10.03	n/a	n/a	78.67 ± 11.35	n/a	n/a	n/a	n/a	n/a	n/a	Significant improvement on modified BI only
		47.08 ± 9.90		12 treatment				n/a	n/a	48.75 ± 10.57	n/a	n/a	69.75 ± 9.90	n/a	n/a	n/a	n/a	n/a	n/a	
Prasad et al. (2012) [22]	Non-RCT	54 (38–70)	11 MCA	11 treatment	Autologous bone marrow-derived MSC	40 × 10^6^	Intravenous	12.27 ± 5.16	3.45 ± 1.04	34.09 ± 22.23	4.80 ± 5.47	2.09 ± 1.30	79.09 ± 20.23	n/a	n/a	n/a	n/a	n/a	n/a	Significant neurological outcome in the subacute stroke
Jiang et al. (2013) [23]	Prospective cohort	48.5 (40–59)	4 MCA	4 treatment	Umbilical cord-derived MSC	20 × 10^6^	Intra-arterial	n/a	4.00 ± 0.816	n/a	n/a	3.25 ± 0.50	n/a	n/a	n/a	n/a	n/a	n/a	n/a	Small size of samples; significant improvement
Banerjee et al. (2014) [24]	Non-RCT	57 (45–75)	4 MCA 1 PCA	5 treatment	CD34 ± stem cell	2.42 (1.2–2.79) × 10^6^	Intra-arterial	10.40± 5.13	3.80 ± 0.84	n/a	2.20 ± 1.92	1.60 ± 1.14	n/a	n/a	n/a	n/a	n/a	n/a	n/a	Significant improvement
Chen et al. (2014) [25]	RCT	52.8 ± 9.0	30 MCA	15 control	PBSC	(3–8) × 10^6^	Intracerebral	9.60 ± 1.30	2.80 ± 0.40	n/a	9.40 ± 1.20	2.70 ± 0.50	n/a	8.70 ± 1.90	2.70 ± 0.50	n/a	n/a	n/a	n/a	Significant clinical outcome improvement
		50.1 ± 7.7		15 treatment				9.30 ± 0.50	2.90 ± 0.30	n/a	6.70 ± 1.70	2.50 ± 0.50	n/a	5.50 ± 1.80	2.10 ± 0.30	n/a	n/a	n/a	n/a	
Prasad et al. (2014) [26]	RCT	18–75		60 control	Autologous bone marrow mononuclear stem cell	280.75 × 10^6^	Intravenous	11.00 ± 4.44	n/a	n/a	n/a	n/a	n/a	n/a	n/a	n/a	n/a	n/a	n/a	Insignificant results
			108 MCA 5 ACA 7 MCA+ACA	60 treatment				13.00 ± 4.44	n/a	n/a	n/a	n/a	n/a	n/a	n/a	n/a	n/a	n/a	n/a	
Qiao et al. (2014) [27]	Prospective cohort	61.5 (45–85)	5 MCA 1 ACA	6 treatment	Umbilical cord mesenchymal stem cell	0.5 × 10^6^/kgbw	Intravenous	8.17 ± 5.84	4.00 ± 1.10	40.83 ± 33.38	n/a	n/a	n/a	n/a	n/a	n/a	n/a	n/a	n/a	Improved neurological function
Kalladka et al. (2016) [28]	Non-RCT	78 (68–82)	9 MCA 1 MCA+ACA 1 PCA	3 (2 m) *	Human neural stem cell (CTX0E03)	2 × 10^6^	Intracerebral	7.67 ± 1.53	4.00 ± 0.00	11.00 ± 1.00	5.67 ± 2.08	n/a	n/a	4.33 ± 2.08	n/a	12.67 ± 1.15	5.67 ± 1.15	n/a	12.00 ± 1.73	Improved neurological function; no controls
		69 (61–75)		3 (5 m) *		5 × 10^6^		8.00 ± 2.00	3.67 ± 0.58	11.67 ± 2.52	6.33 ± 3.06	n/a	n/a	6.33 ± 2.89	n/a	14.67 ± 2.08	5.67 ± 4.04	n/a	14.33 ± 3.51	
		64 (60–68)		3 (10 m) *		10 × 10^6^		7.33 ± 0.58	2.67 ± 0.58	14.33 ± 1.53	4.33 ± 0.58	n/a	n/a	4.67 ± 1.15	n/a	14.67 ±3.21	4.00 ± 1.73	n/a	13.33 ± 1.53	
		66 (61–71)		2 (20 m) *		20 × 10^6^		6.50 ± 0.71	3.00 ± 0.00	13.50 ± 2.12	3.50 ± 2.12	n/a	n/a	3.50 ± 3.53	n/a	16 ± 2.83	4.00 ± 0.00	n/a	17.5 ± 3.53	
Hess et al. (2017) [29]	RCT	18–33	n/a	63 control	Multipotent adult progenitor cell	(400–1200) × 10^6^	Intravenous	13.40 ± 3.70	n/a	n/a	n/a	n/a	n/a	n/a	n/a	n/a	n/a	n/a	n/a	Insignificant results
				71 treatment				13.30 ± 3.50	n/a	n/a	n/a	n/a	n/a	n/a	n/a	n/a	n/a	n/a	n/a	
Jin et al. (2017) [30]	RCT	53.10 ± 13.07	13 anterior circulation 7 posterior circulation	10 control	Autologous bone marrow–derived MSC	10 × 10^6^	Intracerebral	10.70 ± 3.71	4.10 ± 0.99	15.00 ± 8.50	8.20 ± 3.49	3.90 ± 1.10	29.00 ± 12.87	6.50 ± 3.34	3.40 ± 0.97	41.5 ± 17.65	5.70 ± 3.12	3.10 ± 1.10	47.00 ± 24.06	Lumbar subarachnoid injection; significant neurological improvement
		50.80 ± 17.43		10 treatment				12.30 ± 3.95	4.60 ± 0.70	14.50 ± 13.01	9.40 ± 3.81	4.00 ± 0.82	26.00 ± 16.80	8.80 ± 3.71	3.60 ± 0.70	37.5 ± 15.86	8.60 ± 3.69	3.00 ± 1.333	51.50 ± 26.15	
Laskowitz et al. (2018) [31]	Non-RCT	65.5 (45–79)	10 MCA	10 treatment	Umbilical cord blood stem cell	1680 (840–2920) × 10^6^	Intravenous	11.20 ± 1.62	4.40 ± 0.52	18.80 ± 12.26	n/a	n/a	n/a	n/a	n/a	n/a	n/a	n/a	n/a	Significant improvement
Jailllard et al. (2019) [12]	RCT	53	n/a	15 control	Autologous bone marrow–derived MSC	(100–300) × 10^6^	Intravenous	12.75 ± 1.50	4.00 ± 0.00	42.50 ± 14.51	9.40 ± 4.70	3.00 ± 0.66	77.86 ± 25.40	n/a	n/a	n/a	8.43 ± 4.96	3.07 ± 1.10	85.00 ± 20.48	Insignificant clinical outcome, except for the motoric score
				16 treatment				13.5 ± 2.46	3.875 ± 0.16	45.00 ± 18.82	8.94 ± 5.20	3.00 ± 0.63	80.63 ± 30.87	n/a	n/a	n/a	7.73 ± 5.78	2.75 ± 0.93	82.00 ± 27.83	
Savitz et al. (2019) [14]	RCT	60.7 ± 10.4	n/a	19 control	Autologous bone marrow–derived ALD-401	3.8 × 10^6^	Intra-arterial	n/a	n/a	n/a	n/a	n/a	n/a	n/a	n/a	n/a	n/a	n/a	n/a	No significant improvement of the neurofunctional outcome between groups
				20 treatment																
Steinberg et al. (2019) [3]	Non-RCT	64 (33–75)	n/a	6 (2.5 m) *	Modified bone marrow MSC (SB623)	2.5 × 10^6^	Intracerebral	9.30 ± 1.70	3.22 ± 0.43	n/a	n/a	n/a	n/a	n/a	n/a	n/a	n/a	n/a	n/a	Significant improvement of NIHSS score; insignificant result of mRS
				6 (5 m) *		5 × 10^6^														
				6 (10 m) *		10 × 10^6^														
Vahidy et al. (2019) [13]	Non-RCT	63.7 ± 12.5	n/a	185 control	Autologous bone marrow–derived MSC	10 × 10^6^/kgbw	Intravenous	n/a	0.40 ± 0.85	n/a	n/a	n/a	n/a	n/a	n/a	n/a	n/a	n/a	n/a	Favorable safety
		60.7 ± 13.3		25 treatment				n/a	0.08 ± 0.40	n/a	n/a	n/a	n/a	n/a	n/a	n/a	n/a	n/a	n/a	
Zhang et al. (2019) [11]	Prospective cohort	42 (30–49)	n/a	3 (12 m) *	Neural stem cell (NSI-566)	12 × 10^6^	Intracerebral	5.33 ± 3.51	n/a	n/a	n/a	n/a	n/a	n/a	n/a	n/a	n/a	n/a	n/a	Significant improvement; imaging revealed new neural tissue formation
		43 (41–45)		3 (24 m) *		24 × 10^6^		7.67 ± 2.08	n/a	n/a	n/a	n/a	n/a	n/a	n/a	n/a	n/a	n/a	n/a	
		48 (37–54)		3 (72 m) *		72 × 10^6^		6.00 ± 1.00	n/a	n/a	n/a	n/a	n/a	n/a	n/a	n/a	n/a	n/a	n/a	
Chung et al. (2021) [32]	RCT	64.27 ± 13.25	n/a	15 control	Autologous bone marrow–derived MSC	1 × 10^6^/kgbw	Intravenous	14.47 ± 5.32	4.47 ± 0.83	19.80 ± 25.5	n/a	n/a	n/a	n/a	n/a	n/a	n/a	n/a	n/a	Insignificant overall results; significant improvement in lower extremity motor function
		63.03 ± 14.36		39 treatment				11.36 ± 5.20	4.26 ± 0.75	28.28 ± 26.63	n/a	n/a	n/a	n/a	n/a	n/a	n/a	n/a	n/a	
Kang Law et al. (2021) [33]	RCT	64.0 ± 13.9	17 MCA	9 control	Autologous bone marrow–derived MSC	2 × 10^6^/kgbw	Intravenous	n/a	n/a	n/a	n/a	n/a	n/a	n/a	n/a	n/a	n/a	n/a	n/a	Significant BI improvement compared with the control group
		54.60 ± 13.2		8 treatment				n/a	n/a	n/a	n/a	n/a	n/a	n/a	n/a	n/a	n/a	n/a	n/a	
Ruiz et al. (2022) [34]	RCT	76 (69–80)	19 MCA	10 control	Adipose-derived MSC	1 × 10^6^/kgbw	Intravenous	n/a	n/a	n/a	n/a	n/a	n/a	n/a	n/a	n/a	n/a	n/a	n/a	No significant neurological improvement between the treatment groups
		78 (70–82)		9 treatment				n/a	n/a	n/a	n/a	n/a	n/a	n/a	n/a	n/a	n/a	n/a	n/a	

RCT, randomized controlled trial; MSC, mesenchymal stem cell; PBSC, peripheral blood stem cell; NIHSS, National Institutes of Health Stroke Scale; mRS, modified Rankin scale; BI, Barthel index; * in a million cells.

## 3. Results

A total of 175 studies were identified and screened. Of these, thirty-three were assessed for eligibility and twenty-one (eleven PubMed studies, seven Scopus studies, and three Google Scholar studies) were included in the meta-analysis (Table 2).

### 3.1. Demographics, Timing of Intervention, Territory of Stroke, and Type of Stem Cell

The included studies were conducted in Asia, the United States, and Europe. Most of the studies were from Asia (12 out of 21; 57.1%), mainly from China (5 out of 21; 23.8%). Five (23.8%) studies were conducted in the United States, while the studies in Europe (5 out of 21; 23.8%) were undertaken primarily in the United Kingdom (3 out of 21; 14.2%).

Across the 21 studies, 836 patients were included, with a median age of 60.6 years (range 30–85 years). The stem cell therapy group consisted of 406 patients; 247 (60.83%) participants were male and 159 (39.16%) were female. Two of twenty-one studies defined intravascular tPA and endovascular thrombectomy as the intervention for the participants [14,29]. In contrast, other studies described a general supportive therapy for stroke (e.g., antiplatelet, antihypertensive, and rehabilitation) [12,25,30]. One study described the premedications given before stem cell administration (diphenhydramine, hydrocortisone, acetaminophen) but not specifically for the stroke [31].

A total of 390 comorbidities were reported. Hypertension was the most common, in 118 (29.06%) out of the 390 participants; 80 (19.7%) participants were smokers; and diabetes was reported in 68 (16.74%) participants, dyslipidemia in 70 (17.24%), and cardiac problems in 54 (13.3%).

The duration from the onset of stroke and stem cell administration was reported in 333 (82.02%) out of the 406 participants. Most participants (35.22%) had stem cell therapy in the subacute phase, followed by chronic stroke, with 108 (26.6%) participants, while the remaining had acute stroke (20.2%).

The area of vascularization of strokes was reported for 339 (83.49%) out of 406 participants. Most strokes had middle cerebral artery (MCA) involvement (87.6%), followed by anterior cerebral artery (ACA) and MCA involvement (2.4%), while posterior circulation and ACA strokes each occurred in 2.09% and 1.8%, respectively.

Six different stem cell types and sources were administered, namely bone marrow, peripheral blood, umbilical blood, adult progenitor cells, and human neural stem cells. Most of the participants had bone marrow-derived stem cell therapy (265 participants, 65.27%), followed by multipotent stem cells (71, 17.49%), and neural-derived peripheral and umbilical blood-derived stem cells were each administered to 20 (4.92%) participants. In the most recent study, adipose-derived stem cells were used in nine (2.21%) participants (Table 3).

### 3.2. Route of Administration: Clinical Outcomes and Adverse Events

The intravenous group were treated with the most used route of administration and consisted of 304 (78.87%) participants. The intracerebral group consisted of 64 (15.76%) participants, while the intra-arterial group consisted of 38 (9.35%) participants.

#### 3.2.1. Clinical Outcome

The clinical outcome data that we extracted were 6 months (6 of 21 studies), 12 months (4 of 21 studies), and 24 months NIHSS (3 of 21 studies); 6 months, 12 months, and 24 months mRS (2 of 21 studies each); 6 months, 12 months, and 24 months BI (4 of 21, 2 of 21, and 3 of 21 studies, respectively). The mean baseline NIHSS score showed wide variation, namely 9.03 ± 1.76 in the intracerebral group, 10.4 ± 5.13 in the intra-arterial group, and 12.20 ± 4.10 in the intravenous group. We failed to extract some of the data due to the reported measures in some articles only being reported as median values. Baseline mRS scores were not used as a variable, with 3.36 ± 0.38 in the intracerebral group, 3.89 ± 0.83 in the intra-arterial group, and 3.30 ± 0.65 in the intravenous group. The baseline BI score was 13.48 ± 7.12 in the intracerebral group, with no available data for processing in the intra-arterial group, and 34.21 ± 21.64 in the intravenous group.

The NIHSS clinical outcomes after 6 months showed a decrease of 6.96 ± 2.36 in the intracerebral group, 2.2 ± 1.92 in the intra-arterial group, and 7.25 ± 5.31 in the intravenous group. The mRS score after 6 months also decreased by 3.34 ± 0.63 in the intracerebral group, 2.33 ± 0.86 in the intra-arterial group, and 2.63 ± 0.90 in the intravenous group. The improvement in BI score was marked by an increase after 6 months, with 26 ± 16.80 in the intracerebral group and 76.85 ± 21.41 in the intravenous group, while there were no available data in the intra-arterial group.

There was a tendency of decreasing NIHSS and mRS scores and increasing BI scores, indicating better clinical outcomes, after 12 and 24 months in the stem cell therapy group (Table 4).

#### 3.2.2. Adverse Events (Based on Route of Administration)

AEs and SAEs were defined by the terms of the Common Terminology Criteria for Adverse Events (CTCAE) version 5.0. The grading of AEs and SAEs also followed CTCAE version 5.0. An SAE was defined as CTCAE grade 3 or more [35]. AEs were reported to have occurred 431 times in 21 studies, while SAEs were reported 101 (26.09%) times. The numbers varied across the different routes of administration. 

AEs in the intravenous group occurred in 282 (65.42%) patients, with 68 (24.11%) classified as severe, comprising 23.05% of the reported patients with AEs in the intravenous group. 

AEs in the intracerebral group occurred in 123 (28.53%) patients, with 28 (22.76%) considered severe, comprising 44.44% of the reported patients with AEs in the intracerebral group.

In the intra-arterial group, AEs occurred in 26 (6.03%) patients, with 5 (19.23%) classified as severe, accounting for 17.24% of the reported patients with AEs in the intra-arterial group. Table 5 and Table 6 provide a complete list of adverse occurrences and a summary of them categorized by route of administration. 

**Figure 2:**
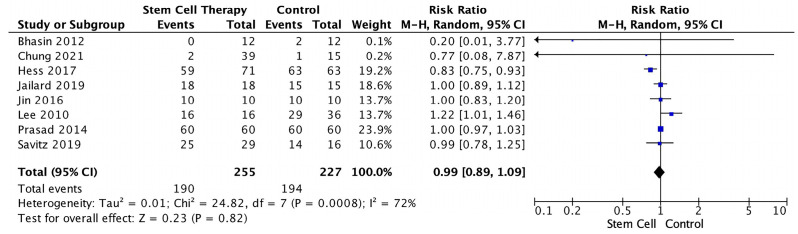
Forest Plot for the Risk of Adverse Events in Stem Cell Therapy [12,14,19,21,26,29,30,32].

Using risk ratio analysis, we compared the stem cell and control groups for AE and SAE risk. This analysis involved eight studies with available data. Hence, the subgroup analysis population was heterogenous (I^2^ = 72%). The results showed the stem cell groups had less risk of AE, although it was not statistically significant (RR = 0.99, 95% CI 0.89–1.09, *p* = 0.82, I^2^ = 72%) (Figure 2).

**Figure 3:**
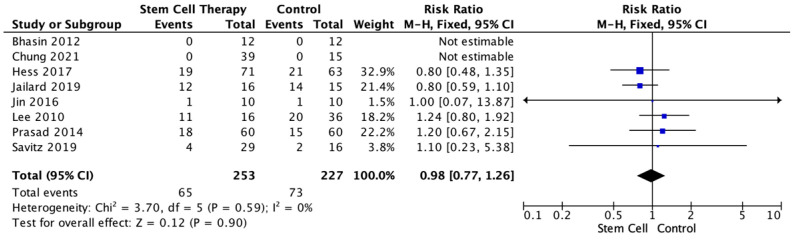
Forest Plot for the Risk of Severe Adverse Events in Stem Cell Therapy [12,14,19,21,26,29,30,32].

We also compared the stem cell and control groups for SAE risk. This analysis involved eight studies with available data. The results showed the stem cell groups had less risk of SAE, but it was not statistically significant (RR = 0.98, 95% CI 0.77–1.26, *p* = 0.90, I^2^ = 0%). The sub-group analysis population was homogenous (I^2^ = 0%) (Figure 3).

We also provide the results of the quality assessment which can be accessed in Supplementary Materials in order to help the reader in the analysis of the results of the manuscript.

## 4. Discussion

### 4.1. Demographics, Timing of Intervention, Territory of Stroke, and Type of Stem Cell

Men outnumbered women by a ratio of 1.65:1, given that men have a greater age-adjusted stroke incidence; however, women are known to have a higher risk of stroke due to their longer life expectancy [36,37]. These findings may have been affected by the choice of therapy for different genders. Some studies reported worse clinical outcomes with mechanical thrombectomy in women [38,39,40]. 

Hypertension, a risk factor for ischemic and hemorrhagic stroke [41], was the most prevalent comorbidity, followed by diabetes, dyslipidemia, and cardiac issues with a significant role on the stroke outcome [42]. 

By the time stem cell therapy was administered, most individuals had had subacute strokes. The subacute period of stroke is considered the ideal time for the brain to self-repair [14]. Chronic strokes, on the other hand, necessitate the opening of the blood–brain barrier (BBB) to facilitate a more successful treatment strategy. Previous research has shown that manipulating the BBB with IV mannitol prior to stem cell treatment results in an increase in trophic factors in the brain following infarction; however, the mRS score was not observed [32,43]. It should be noted that the increased permeability of the BBB could worsen the ischemic process through increased inflammatory factors at the injury site [44]. On the other hand, stem cell treatment for acute stroke has been shown to lower the inflammatory response, enhancing the tissue repair and neuroprotection processes [45,46]. 

According to the available data, the MCA accounts for the most stroke pathology [47]. Meanwhile, isolated ACA territory strokes are rare, reported to only account for 0.5–3% of all ischemic strokes [48,49]. Only 1.8% of patients in our research experienced an isolated ACA stroke, whereas 2.4% had both MCA and ACA stroke. Only two trials in this evaluation reported on patients who had received stem cell treatment for posterior circulation strokes [30]. Histological differences also play a role. The brainstem is composed more of white than gray matter [50], and microglia, along with the precursors of oligodendrocytes, are known to help secrete trophic factors [51].

Most studies utilized autologous bone marrow–derived stem cells [3,12,13,14,19,20,21,22,26,30,32,46]. The use of these stem cells to mend neural tissue was described with encouraging results [52,53,54,55,56] and reportedly showed only motoric improvement [12]; in addition, Prasad observed a slight improvement in outcomes [26]. Lee observed considerable improvement in the early post-therapeutic period but no improvement at later stages [19]. Kalladka displayed improved neurological functions, although no comparative controls were employed in that study [28].

### 4.2. Route of Administration: Clinical Outcome and Adverse Events

The route of administration is one of the most contentious issues, as it relates to the efficacy and safety of the procedure on patients [46,57]. This is discussed in more depth in our study.

#### 4.2.1. Intravenous Route

Despite being the most frequently utilized approach in the literature analyzed in this investigation, only four out of eleven studies using the intravenous route for stem cell delivery reported favorable clinical results, and only one out of four was an RCT. The other three were not controlled studies, although they all revealed improved neurological outcomes following therapy for varying lengths of time. Apart from the trial by Lee et al. (2018), all previous RCTs failed to demonstrate substantially better neurological results [12,26,29,32].

The intravenous route is regarded as the most straightforward and the least invasive technique available [58], compared with its intravascular counterpart, the intra-arterial route. The most recent literature revealed that intravenous stem cell treatment for chronic ischemic stroke had unsatisfactory results [32].

On the other hand, intravenous stem cell treatment causes stem cells to migrate to organs other than the brain due to their systemic nature. Research has indicated that time is critical in stroke therapies, particularly for acute strokes [59], and the fact that brain tissues are very vulnerable to hypoxia [60]. Hess reported that the early administration of allogeneic multipotent adult stem cells (<36 h) in cases of acute stroke is associated with better clinical outcomes [29].

The impact of graft time and route on the survival and functional advantages of CD133+ human bone marrow cells in a stroke model might help personalize transplantation methods for individual instances [61]. Localized graft survival with improvement in motor deficits was observed in both immediate and delayed intracerebral transplantation; however, compared with graft survival, behavioral improvement was only observed in immediate intravenous transplantation [61].

As previously noted, stroke is accompanied by severe inflammation and an immune response early in the disease process; therefore, employing an invasive procedure in the acute stage may not be advised [62]. Consequently, in the early stage, pursuing intravenous transplantation, a minimally invasive treatment, is more advisable.

#### 4.2.2. Intra-Arterial Route

One RCT trial conducted using the intra-arterial route was found to have unsatisfactory results [14], while the other two studies lacked controls [23,24].

Intra-arterial stem cell administration for stroke facilitates a smaller dose and more concentrated delivery of cells to the cerebral lesion [60] Savitz mentioned that the therapy’s dosage has no correlation with the neurofunctional outcome [14]. Moreover, intra-arterial administration requires a smaller number of cell grafts, compared with the intravenous route [63], and Zhang et al. (2018) reported better neurologic outcomes in the intra-arterial group compared with the intravenous and intracerebral routes using an animal model [58].

In general, stem cell delivery via the intra-arterial route has a mechanism of action similar to stem cell administration via the intravenous route [8]. The primary method of healing following stem cell administration via the intra-arterial route is via the stimulation of injected stem cell to produce growth factors, cytokines, and chemokines by paracrine stem cells that contribute to anti-apoptotic effects, angiogenesis, and neurogenesis [64].

#### 4.2.3. Intracerebral Route

All five studies reported a better clinical outcome regarding intracerebral administration [25,30], and one reported insignificant improvement in the 24-month mRS, while there were improvements in the alternative scoring systems [3].

Intracerebral stem cell treatment in instances of cerebral ischemia provides the benefit of minimizing concern for biodistribution and focused migration of cells into infarcted neural tissue [58]. Additionally, it has the advantage of direct inoculation and targeted therapy to the infarcted cerebral tissue without the disadvantages of cell dispersion and ineffective localization associated with the intravenous route and microembolization and bubble formation associated with the intra-arterial route [65].

The mechanism of the action of stem cells administered via the intracerebral route may differ from the intravascular one, as it directs the stem cell delivery [61]. Since the survival rate and total number of new neurons are exceedingly low, the intracerebral route may provide neuronal healing. Since the BBB is disturbed in stroke, transplanted stem cells easily traverse the BBB, congregating in infarcted brain regions and re-establishing the BBB’s integrity [10,66,67,68,69].

Some reports demonstrated that the intracerebral route may decrease apoptosis in the ischemia border area and be related to remarkable neurological recovery in animal models [10,66,70]. It was reported that human bone marrow mesenchymal stem cell (hMSCs) could reduce the apoptosis in neuronal cell death on cerebral ischemia [68] by secreting a wide range of anti-inflammatory cytokines [67].

However, the invasive nature of this intracerebral delivery technique requires further attention and patient selection. Several studies reported procedure-related adverse events. Kalladka reported one symptomatic procedure-related extradural hematoma and one symptomatic anticoagulant-related subdural hemorrhage event [28]. Steinberg reported one asymptomatic procedure-related subdural hemorrhage event with good recovery [3]. Zhang reported one asymptomatic microcerebral hemorrhage without sequelae [58]. The intracerebral route can be performed using less invasive techniques, such as intraventricular and subarachnoid administration [69]. The subarachnoid route was reported in an RCT and had remarkable clinical outcomes in the experimental group [30].

To the authors’ knowledge, there is no research comparing the efficacy of various modalities of stem cell treatments for ischemic strokes in humans. However, Zhang et al. (2018) reported that the intra-arterial route had superior efficacy compared with other routes (intravenous and intracerebral) in an animal model of cerebral ischemia [58].

Other routes worth noting are intraperitoneal and intranasal administration [69]. These two routes still require further experimental studies to demonstrate their feasibility, safety, and effectiveness. The intranasal route could offer a less invasive approach and is promising as it bypasses the BBB [71].

#### 4.2.4. Adverse Events

The majority of AEs (65.42%) occurred in the intravenous group, while 28.53% occurred in the intracerebral group and 6.03% in the intra-arterial group. The authors recognize that these data should be treated with caution, as they do not consider the number of participants and are only a rudimentary representation of all occurrences. As mentioned above, Laskowitz reported that most AE occurrence was not related to the treatment group (99.1%) [31]. Consistent with this, Vahidy also reported that there were no study-related SAEs in the therapy group [13].

The ratio of all reported SAEs to all reported AEs was 26.09%; however, this figure should be regarded cautiously because not all studies reported on all types of AE [19,28]. The intracerebral group had the highest rate of SAEs (44.44%), followed by the intravenous group (23.05%), while the intra-arterial group had the lowest rate of SAEs (17.24%). These findings might be due to the invasiveness of the intracerebral route and the limited sample in the intra-arterial group [28].

The comparison of AEs and SAEs between the stem cell and control groups was also studied using forest plot. Although not statistically significant, AEs and SAEs occurred less in the stem cell group. These findings might have been caused by patients’ comorbidities.

## 5. Conclusions

In conclusion, although stem cell treatment demonstrated superior results over standard conservative therapy alone in stroke patients, our data show that several factors (e.g., patient’s comorbidities, treatment’s timing, administration route) might blur the treatment’s benefits and safety. To the best of our knowledge, this review is the first study that determines the functional outcome and the treatment’s adverse events based on the delivery route. The findings of trials utilizing various delivery methods demonstrated positive effectiveness and safety. Although intracerebral injection resulted in better neurological outcomes than other routes, it was associated with a higher rate of AE because of its intrusive nature. On the other hand, the intra-arterial and intravenous routes had unsatisfactory outcomes but the highest degrees of safety, although the most AE occurrences were not related to the treatment protocols. Since we found that the studies’ outcomes and follow ups are both varied and limited in most of the included studies in this review, a more extensive and focused investigation is required to evaluate the effectiveness and safety of this future treatment strategy.

## Figures and Tables

**Table 1:** PICOS (Population, Intervention, Comparison, Outcome Measures, and Study Design) model.

PICOS Item	Inclusion Criteria	Exclusion Criteria
Population	Adult ischemic stroke patients receiving stem cell therapy Stem cell therapy via intracerebral, intraventricular, subarachnoid, intra-arterial, intravenous, intraperitoneal, or intranasal administration	Child ischemic stroke patients Traumatic brain injury Hemorrhagic stroke
Intervention	Stem cell therapy via intracerebral administration	
Comparison	Stem cell therapy via intraarterial administration Stem cell therapy via intravenous administration Control or sham group	
Outcome measures	Clinical outcomes were measured using the modified Rankin scale (mRS) The National Institute of Health Stroke Scale (NIHSS) The Barthel index (BI). The adverse events (AE) Severe adverse events (SAE)	
Study design	Randomized clinical trials Non-randomized clinical trial Pilot randomized trials Retrospective analyses	Reviews Letters to the editor Abstracts
Publication	Published in English Access to full text	Unpublished studies Study protocols

**Table 3:** Demographic Characteristics of the Studies.

Description		Number, n (%)
Total patients	n	817 participants
Median age	Median (min–max)	60.6 years (30–85)
Gender		
Male	n	247 participants (60.83%)
Female	n	159 participants (39.16%)
Comorbidities		
Hypertension	n	118 participants (29.06%)
Diabetes mellitus	n	68 participants (16.74%)
Dyslipidemia	n	70 participants (17.24%)
Cardiac problems	n	54 participants (13.3%)
Smoking	n	80 participants (19.7%)
Standard treatment modalities		
Tissue Plasminogen Activator (TPA)	n	2 studies
Mechanical revascularization	n	2 studies
Patients with stem cell therapy	n	406 participants (49.7%)
Based on the route of administration		
Intracerebral group (5 studies)	n	64 participants (15.76%)
Intra-arterial group (3 studies)	n	38 participants (9.35%)
Intravenous group (13 studies)	n	304 participants (74.87%)
Based on stroke onset		
Acute (1–7 days)	n	82 participants (20.2%)
Subacute (1–3 weeks)	n	143 participants (35.22%)
Chronic (>3 weeks)	n	108 participants (26.6%)
Based on stem cell source		
Bone marrow	n	265 participants (65.27%)
Peripheral blood	n	20 participants (4.92%)
Umbilical blood	n	20 participants (4.92%)
Multipotent stem cell	n	71 participants (17.49%)
Neural stem cell	n	20 participants (4.92%)
Adipose	n	9 participants (2.21%)
Based on stroke territory		
Anterior Cerebral Artery (ACA) and Middle Cerebral Artery (MCA)	n	8 participants (2.4%)
ACA	n	6 participants (1.8%)
MCA	n	297 participants (87.6%)
Anterior circulation	n	13 participants (3.8%)
Posterior circulation	n	7 participants (2.09%)

**Table 4:** Clinical Outcomes.

Description		Number n (%)
Clinical outcome baseline		
NIHSS		
Intracerebral group	Mean ± SD	9.03 ± 1.76
Intra-arterial group	Mean ± SD	10.4 ± 5.13
Intravenous group	Mean ± SD	12.20 ± 4.10
mRS		
Intracerebral group	Mean ± SD	3.36 ± 0.38
Intra-arterial group	Mean ± SD	3.89 ± 0.83
Intravenous group	Mean ± SD	3.30 ± 0.65
BI		
Intracerebral group	Mean ± SD	13.48 ± 7.12
Intra-arterial group	Mean ± SD	n/a
Intravenous group	Mean ± SD	34.21 ± 21.64
Clinical outcome after 6 months		
NIHSS		
Intracerebral group	Mean ± SD	6.96 ± 2.36
Intra-arterial group	Mean ± SD	2.2 ± 1.92
Intravenous group	Mean ± SD	7.25 ± 5.31
mRS		
Intracerebral group	Mean ± SD	3.34 ± 0.63
Intra-arterial group	Mean ± SD	2.33 ± 0.86
Intravenous group	Mean ± SD	2.63 ± 0.90
BI		
Intracerebral group	Mean ± SD	26 ± 16.80
Intra-arterial group	Mean ± SD	n/a
Intravenous group	Mean ± SD	76.85 ± 21.41
Clinical outcome after 12 months		
NIHSS		
Intracerebral group	Mean ± SD	6.21 ± 2.49
Intra-arterial group	Mean ± SD	n/a
Intravenous group	Mean ± SD	1.58 ± 2.02
mRS		
Intracerebral group	Mean ± SD	2.7 ± 0.46
Intra-arterial group	Mean ± SD	n/a
Intravenous group	Mean ± SD	n/a
BI		
Intracerebral group	Mean ± SD	25.38 ± 8.74
Intra-arterial group	Mean ± SD	n/a
Intravenous group	Mean ± SD	n/a
Clinical outcome after 24 months		
NIHSS		
Intracerebral group	Mean ± SD	6.67 ± 4.03
Intra-arterial group	Mean ± SD	n/a
Intravenous group	Mean ± SD	7.73 ± 5.78
mRS		
Intracerebral group	Mean ± SD	3 ± 1.33
Intra-arterial group	Mean ± SD	n/a
Intravenous group	Mean ± SD	2.75 ± 0.93
BI		
Intracerebral group	Mean ± SD	31.86 ± 13.76
Intra-arterial group	Mean ± SD	n/a
Intravenous group	Mean ± SD	82 ± 27.83

**Table 5:** Detailed Adverse Events for Each Included Study.

Author(s)	Patients	Sample Size	Route of Administration	AE	SAE (CTCAE 3 or More)	SAE Details
Jailllard et al. (2019) [12]	31	15 control	Intravenous	24	14	1 death, 1 recurrent stroke, 2 humeral fracture, 5 epileptic, 3 pneumonia, 1 gastrostomy, 1 atrial flutter
		16 treatment		18	12	2 depression, 1 humeral fracture, 6 epileptic, 1 DVT, 2 pneumonia
Lee et al. (2010) [19]	52	36 control	Intravenous	29	20	1 new onset stroke, 2 angina, 9 pneumonia, 1 acute kidney injury, 1 systemic cancer, 1 benign mass, 5 seizure
		16 treatment		18	11	2 new onset strokes, 1 angina, 1 PAOD, 3 pneumonia, 1 benign mass, 3 seizure
Honmou et al. (2011) [20]	12	12 treatment	Intravenous	6	0	None
Bhasin et al. (2012) [21]	24	12 control	Intravenous	2	0	None
		12 treatment		0	0	
Prasad et al. (2012) [22]	11	11 treatment	Intravenous	0	0	None
Jiang et al., 2013 [23]	4	4 treatment	Intra-arterial	n/a	0	None
Banerjee et al. (2014) [24]	5	5 treatment	Intra-arterial	1	1	1 pneumonia
Chen et al. (2014) [25]	30	15 control	Intracerebral	n/a	0	None
		15 treatment		n/a	0	
Prasad et al. (2014) [26]	120	60 control	Intravenous	60	15	1 hypotension, 1 pneumonia, 1 fracture in lower limb, 5 death, 7 CNS AE
		60 treatment		61	18	1 pneumonia, 1 PAOD, 2 fractures in the lower limb, 8 death, 6 CNS AE
Qiao et al. (2014) [27]	6	6 treatment	Intravenous	5	0	4 fever, 1 dizziness
Kalladka et al. (2016) [28]	11	3 (2 m)	Intracerebral	n/a	16	1 subdural hematoma, 1 epidural hematoma, 1 stroke, 1 cystoscopy, 2 bleed on burrhole site, 1 malignant melanoma, 5 gastrointestinal AE, 1 seizure, 1 alcohol withdrawal syndrome, 1 collapse, 1 community-acquired pneumonia
		3 (5 m)				
		3 (10 m)				
Hess et al. (2017) [29]	134	2 (20 m)	Intravenous	64	21	6 severe, 6 LT, 9 deaths
		71 treatment		59	19	11 severe, 3 LT, 5 deaths
Jin et al. (2017) [30]	20	10 control	Intracerebral	12	1	1 death due to large infarction
		10 treatment		12	1	1 pneumonia
Laskowitz et al. (2018) [31]	10	10 treatment	Intravenous	113	8	112 AEs were unrelated to the treatment group
Savitz et al. (2019) [15]	48	19 control	Intra-arterial	14	2	1 new onset stroke, 1 astrocytoma,
		20 treatment		25	4	1 muscular pain, 1 UTI, 1 embolism, 1 brain edema
Steinberg et al. (2019) [3]	18	6 (2.5 m)	Intracerebral	20	9	1 seizure, 1 stenting of the carotid artery, 1 asymptomatic subdural hygroma, 1 TIA, 1 hypesthesia, 1 dysphagia, 1 UTI, 1 sepsis, 1 pneumonia
		6 (5 m)		31		
		6 (10 m)		25		
Zhang et al. (2019) [11]	9	3 (12 m)	Intracerebral	18	2	2 cholecystitis
		3 (24 m)		2	0	None
		3 (72 m)		15	0	None
Vahidy et al. (2019) [13]	210	185 control	Intravenous	227	24	No study-related SAE in the therapy group
		25 treatment				
Chung et al. (2021) [32]	54	15 control	Intravenous	1	0	None
		39 treatment		2	0	None
Kang Law et al. (2021) [33]	17	9 control	Intravenous	2	2	No study-related SAE in the therapy group
		8 treatment		2	2	
Ruiz et al. (2022) [34]	19	10 control	Intravenous	12	12	No study-related SAE in the therapy group
		9 treatment		0	0	

CNS, central nervous system; LT, life-threatening; PAOD, peripheral artery occlusive disease; TIA, transient ischemic attack; UTI, urinary tract infection.

**Table 6:** Adverse Events by Stem Cell Route of Administration.

Route of Administration	Number of Patients in Studies Reporting AEs	Number of AEs	Number of SAEs	SAEs/Total Patients
Intravenous	295	282	68	23.05%
Intra-arterial	29	26	5	17.24%
Intracerebral	63	123	28	44.44%
Total	387	431	101	26.09%

## Data Availability

No new data were created or analyzed in this study. Data sharing is not applicable to this article.

## Supplementary Materials

The following supporting information can be downloaded at: https://www.mdpi.com/article/10.3390/jcm12072735/s1.
