# Supplementary figures and images for: Comparison of the Administration Route of Stem Cell Therapy for Ischemic Stroke: A Systematic Review and Meta-Analysis of the Clinical Outcomes and Safety
Source: J Clin Med. 2023 Apr 6;12(7):2735. doi: 10.3390/jcm12072735 (PMC10094955; doi:10.3390/jcm12072735)

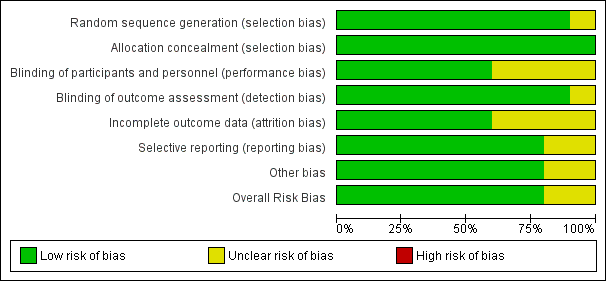

Supplement: Supplementary file 1 [file jcm-12-02735-s001.zip › Rob-2 proportional assessment.png]

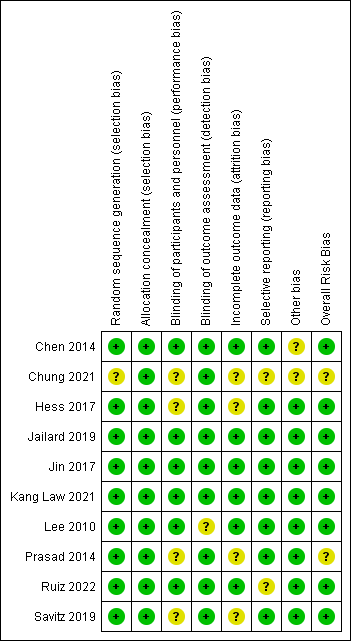

Supplement: Supplementary file 1 [file jcm-12-02735-s001.zip › Rob-2 table.png]

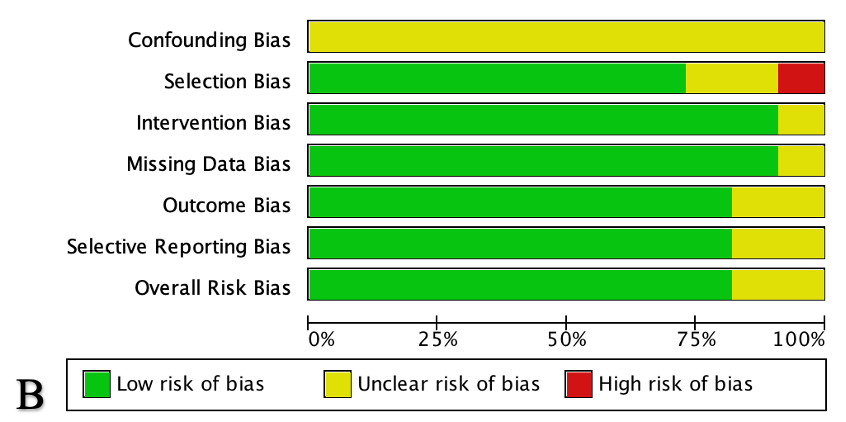

Supplement: Supplementary file 1 [file jcm-12-02735-s001.zip › Robins-1 proportional assessment.png]

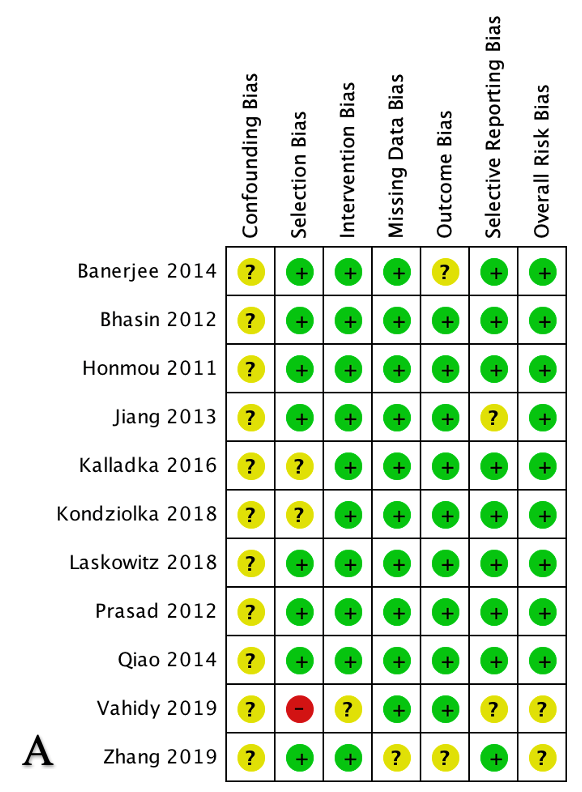

Supplement: Supplementary file 1 [file jcm-12-02735-s001.zip › Robins-1 table.png]
